# Cough and Its Treatment in Pulmonary and Laryngeal Tuberculosis

**Published:** 1901-09

**Authors:** Henry Levien

**Affiliations:** Medical director and physician-in-charge of The Liberty Sanitarium, Liberty, N. Y.


### Cough and Its Treatment in Pulmonary and Laryngeal Tuberculosis.

By HENRY LEVIEN, M. D.,
Medical director and physician-in-charge of The Liberty Sanitarium, Liberty, N. Y.

IN THE way of preface, I will say that my apology for bringing this rather trite subject to the attention of the medical profession, is the opportunity I enjoyed through constant personal observation on fifty patients under my care in the course of the last twelve months. Circumstances brought me in quite close, perhaps too close, contact with the above number of tuberculous patients, while in the capacity of physician-in-charge of a sanitarium for the treatment of persons suffering from pulmonary and laryngeal tuberculosis.

Of all the features, physical and mental, which make up the sum total of a consumptive, cough is the most distressing and prominent symptom, and as such, should engage the mind of the physician to a larger and deeper extent concerning its amelioration and suppression in the treatment of tuberculosis. It is the psychical as well as the physical tormentor of the unfortunate consumptive. It is a well-known fact that tuberculous patients to the exclusion of all others will never admit that they individually are afflicted with this dreadful disease; they are suffering from a "slight cough," which must soon pass away.

"If not for the cough, I would feel perfectly well," says one. "Oh, doctor, if you would only stop my cough, I would not feel sick at all," says another. Hemoptysis, even severe hemorrhages, are quite often forgotten; night sweats, though disagreeable, are seldom the subject of complaints—they would yet consider themselves only victims of a bad cold, which manifests itself in this wretched cough. Though sent by their respective physicians to a sanitarium where only tuberculous patients are treated, yet on entering the institution, they will always ask not to put them in close contact with "consumptives," because they have not this disease; they are only coughing slightly, having contracted a severe cold. This illusion is rather in some cases, especially in incipient tuberculosis very fortunate, as the patient is hopeful of a speedy recovery and struggles energetically for the restoration of his health. Such being the case, our efforts must be directed towards amelioration and suppression of cough, to keep up that favorable illusion.

Again this distressing cough of consumptives, if not checked in some way, wrecks the wasting constitution unmercifully. It deprives them of sleep, which rest is so indispensable for healthy and sick people in general and for those afflicted with tuberculosis in particular.

Cough is very often the cause of retching and vomiting. In the first instance, the afflicted feels wretched; in the second, he is getting exhausted and disgusted with his miserable existence.

Though not by any means the sole cause of pulmonary hemorrhages, yet by causing hyperemia of the lung tissues and at the same time interfering with the normal pulmonary circulation, the venous return, cough thus becomes the executor of pulmonary hemorrhages. Paradoxical as it may sound, yet we would entertain an idea that if pulmonary tuberculosis and phthisis in general could have existed without coughing, hemorrhages would seldom be heard of. So far as our observation goes, hemorrhages seldom arise spontaneously. This may be the case with the first one, the rather unexpected herald of some trouble, but when the disease is more or less advanced, the hacking dry cough is the unwelcome messenger of the "ruby" as sanitarium inmates, or "lungers" are wont to name hemorrhage, in the way of shortening it, we suppose.

Because of this cough many a patient shuns society and as in the case of sanitariums avoids taking their meals in the general dining room, and if not ameliorated, this condition may lead to dejected spirits and even melancholia. There is hardly any difference as to the stage of pulmonary tuberculosis, so far as cough is concerned. They cough in the first stage, more so in the second, when every inspiratory act is a coughing fit, and still more in the third stage, unless the patient by this time was well taken care of, availed himself of the requisite climatic and hygienic surroundings, which might have been the combined factors in drying up the cavity or cavities.

What are the means at our disposal for suppressing this cough? Do the generally administered pulmonary, or as some would have them, gastro-intestinal,antiseptics like creasote and the many coal-tar and sulphur products tend in some way to diminish the cough? In my experience, they do not. Will oils and fats in general do anything towards quieting cough? They may in a general way and in the long run, so to say. That is in addition to plenty of sunshine in the open air, respiratory gymnastics, wholesome and plenty of nourishment, and hydropathic treatment systematically carried on, they may assist in clearing the bronchi and even the alveoli of the superfluous mucopurulent secretions and thus create a feeling of well being, but by the time this is reached, the patient might be considered as one who has fully or nearly recovered from the sickness as a whole and the cough in consequence; but we need something which would act more speedily and in this way hasten the happy outcome.

Inhalations have their sphere of beneficial action upon inflamed and irritated mucous membranes, but the benefit derived from them is quite temporary, and we would not advise this means as a remedial agent to relieve a distressing cough. Inhalations may subserve in "curing" phthisis, with which we are not concerned in this discussion.

Recourse must then be taken in remedies which would act directly and immediately, without at the same time carrying deleterious effects upon the system as a whole.

Hypnotics or nerve sedatives, like chloral, the bromides and the many coal-tar derivatives, which have their especial value in other conditions, should in our opinion never be administered, because intended as they are for quieting the reflexes in certain nervous affections, will not benefit diseases where positive pathological changes are the sole cause of these manifestations. Besides nearly all hypnotics are cardiac depressants, and such remedies should be administered with special caution if given at all, as the heart's action in consumptives is always below par and requires often a whipping up and stimulating.

Resource must be found then in the class of analgesics and anodynes, and then opium in its different forms heads the list. Considering the different preparations and their salts, chronologically so to say, we learn that opium per se is not suitable because of its constipating effect and undesirable sequelæ left after prolonged use.

Dover's powder was at one time an anchor and used extensively. Pulvis ipecac compound, consisting as it does of opium and ipecachuana, is intended to meet two purposes; to cause slight nausea and thus promote expectoration, then to suppress cough by the opium contained therein. We hardly believe it is suitable as a sedative in pulmonary tuberculosis, for in doses which should be effective, the nausea will exceed its limits and cause vomiting, a very undesirable effect, and the opium will then come in too large amounts for the necessary sedation. It may have its place in coryza, probably in influenza, but not in tuberculosis. Morphia would do away with nausea, but the morphia habit and its after-effects should not be lost sight of and therefore should also be relegated to other spheres.

Codeine kept its place in therapeutics as an old tried remedy in suppressing or quieting any kind of coughs. It does so, but the constitution gets so used to its effects when it is administered for any length of time, that we must increase the dosage quite often and extensively.

Heroin is a recent addition to our therapeutic armamentarium. We have given it a thorough trial and found it superior to all previously enumerated drugs. This remedy is an improvement upon and a modification of morphia, especially adapted for the treatment of the respiratory apparatus. It does not replace morphia in all cases; it substitutes it in pulmonary affections only. It has a sedative effect upon the respiratory tract, produces restful sleep, does not constipate, and so far as my experience with the drug goes, does not produce heroin habit. My attention was called a few months ago and urgently requested to give its salt, heroin hydrochlorate, a trial, administering it in a weak solution of hydrochloric acid; I thought it did act in a more efficient way than the heroin plain. But with heroin and heroin hydrochlorate administered *per se*, I had the difficulty in establishing the requisite or standard dosage. Some clinicians have benefited their patients with very small doses as 1-20 of a grain. Tablets are manufactured in the market in doses of 1-12 gr. In our experience neither one nor the other were sufficient to exert the right beneficial effect, and had to order tablets in doses of 1-8 of a grain, administering them one at bed time and during the day *pro re nata*. This seemed to be the proper dose without carrying with it any deleterious after-effect.

In my college days, I heard Prof. Wm. H. Thomson repeatedly urging upon us his opinion that a combination of adequate remedies will act better than any single one given in large doses, and following his advice later in my general practice, I believe I reached my ends better and drugged my patients less, because by combining remedies, each is given in smaller doses, especially when narcotics or other poisons are in question. It was with a two-fold feeling that I met a new remedy, or rather a preparation which is a combination of remedies, destined for the treatment of disease of the respiratory tract. I always had misgivings towards new preparations; but as this preparation, glyco-heroin, seemed to me in no sense a patent medicine, having the contents open before the medical profession, I thought it worthy of trial. The combination which makes up the glyco-heroin should appeal to any one who is treating patients afflicted with pulmonary and laryngeal diseases. The composition of glyco-heroin is in our opinion quite a happy one. Each teaspoonful contains 1-16 gr. of heroin, quite a small dose; hyoscyamus, 1 gr., an adequate addition, as it checks night sweats and acts favorably upon reflexes; ammonium hypophosphite," 3 grs., which is an excellent stimulating expectorant without producing nausea; white pine bark, a tonic and a slight antipyretic), with balsam tolu, glycerin and aromatics. A teaspoonful of this preparation, I found to be a definite dose, the effects of which lasted for three to four hours. Two weeks' trial on six patients convinced me of its utility and superiority over heroin and heroin hydrochlorate, administered *per se.*

My clinical observations in sanitarium treatment of pulmonary and laryngeal diseases cover fifty cases, treated in the course of twelve months, and to make my report on this particu_ lar subject quite comprehensive and convincing, I'll have to depart from the generally accepted mode of tabulation and present it in the shape of three periods, corresponding to the time and number of patients when one or the other remedy was administered.

| Time. 1900. | Number of Patients. | Drug Administered. | Results Observed. |
|---|---|---|---|
| May, June, July, August. | 20 | 7, Pulv. Dover. | Not satisfactory. |
| | | 13, Codeine sulph., ¼ gr. | Effect upon cough favorable, but the doses had soon to be increased. |
| September, October, November and December. | 14 | 8, Heroin, heroin hydro., dose ⅛ gr. in acid water. | Quite favorable, but I always felt that dose was too large. Smaller doses were not effective. |
| | | 6, Glyco-heroin on trial. | |
| 1901. | | | |
| January, February, March, April, May (part). | 16 | Glyco-heroin (Smith) exclusively. | Very favorable. |

We do not deem it necessary to tire the reader with reports of cases in more detail, but we beg to convey our impression that since we started to administer glyco-heroin to our patients as a routine, directing them to take a teaspoonful at bed time and during the day as occasion required—severe cases should take a teaspoonful every two or three hours—a decided improvement in the general well-being of all was felt. In the course of a few weeks, one would hardly believe it was an institution for, or the abode of consumptives, whose business is, so to say, to cough. In the stillness of the night, one could hardly discern any hack or even loose cough, and it convinced us that our patients were enjoying a peaceful and refreshing sleep. It is quite grateful to note that after one week's systematic administration of this remedy in conjunction with out-door life, pulmonary exercises and good dieting, the cough, pain in the chest, pleuritic pains and dyspnea are considerably ameliorated and night sweats entirely disappear. We believe it will not be amiss to cite at least one recent case:

Mr. B., aged 20; New York; law student, previously bookkeeper and salesman. On December, 1900, he caught a cold, coughed slightly; gave it no attention. On February 1, 1901, had an attack of hemoptysis, which lasted but a few days, and but little attention was paid to it. On March 1 he felt pain in the right side of chest. Next day pain increased; had fever. In the evening, a third attack of hemoptysis, and on the third day quite a severe hemorrhage. This kept him in bed for two weeks and in the early convalescence, Dr. M. Moskievitz, his attending physician, sent him to our sanitarium with the history and diagnosis of tubercular pleurisy and consolidation of the right apex anteriorly and posteriorly. Upon microscopical examination of the sputum numbers of tuberculous bacilli were found. He was suffering besides from cardiac debility and dyspnea, and was so weak that two attendants were necessary to bring him up to the sanitarium.

March 30, 9 a.m., examination after one night's rest: Pulse, 120, slightly intermittent; temperature, 97.8°; respiration, 36; weight, 121 lbs., previous weight, 136. Physical examination: on auscultation, right lung, upper two-thirds anteriorly, suppressed respiration and in areas where respiratory sounds were audible, they were bronchial in character. No respiratory sound posteriorly, owing to serofibrinous exudation and thickening of the pleura. Considerable pain in the same side on respiratory effort. Left lung, shallow repressed respiration with moist crepitant and subcrepitant râles. On percussion, dulness under right clavicle, and very dull, approaching flat percussion note posteriorly below the angle of scapula and axillary line. Vocal fremitus increased anteriorly. Heart sounds apparently normal, a state of cardiac palpitation existing. Sputum examination revealed the presence of tubercular bacilli.

Diagnosis: Tuberculosis upper two-thirds of lung anteriorly and posteriorly. Tubercular pleurisy, slight effusion, thickening and recent adhesions of the right side.

Treatment: Rest in bed; windows open day and night. Local treatment consisted in sponging with tepid water, thorough friction, and application of iodine by painting with tincture iodine. Internally, strychnin for the cardiac debility, malt-phosphate as a general tonic and glyco-heroin (Smith) for the painful cough and pain in the right side.

April 7, cough much ameliorated, sleeps well, not disturbed by coughing spells. Pain in the side almost entirely gone. Respiration twenty-four per minute and without panting on walking. Feeling well and strong enough to take short walks without fatigue. No night sweats; pulse, 100; temperature slightly above normal in the afternoon; gained $3\frac{1}{2}$ pounds in weight.

April 14, continues to improve; hardly coughs at all, dyspnea diminished. No night sweats, no hemoptysis; allowed longer walks; physical signs improving. Sleeps well. Weight, 127.

April 21, temperature normal. Quite healthy appearance, able to take gymnastic exercises, heart action much stronger. Appetite good; pain in the side disappeared, though the dullness is still noted. Pulse 96; weight 130 pounds.

April 28, improvement continues; hardly coughs at all. Takes glyco-heroin only at bed time. Left lung entirely clear of râles, consolidated areas of right lung gradually diminishing in size; no pain in the side, though respiratory sounds hardly audible as yet.

May 7, no night sweats; doesn't cough; climbs to top of Walnut mountain without fatigue. Pulse 90, full and regular; weight, 133 pounds. Effusion being absorbed, a feeling of well being prevails.

I had but two occasions to use glyco-heroin in other than tubercular affections, namely asthma. One case was bronchial asthma of a mechanical nature. The patient was a furrier by trade and whenever he would spend a day in his shop, he would get the attack in the evening; morphia hypodermatically, amyl nitrite by inhalation and other remedies would not control these attacks, which would torment him for hours at a time. On one occasion I administered one teaspoonful of glyco-heroin, repeated the dose in ten minutes and the attack was cut short as by magic.

The other case was one of cardiac asthma in a man 72 years old. He gets the attack mostly on retiring or whenever his feet get cold. He is suffering also from arterio-sclerosis and chronic interstitial nephritis. His attacks were also cut short by one or two doses of glyco-heroin, and in this I fully coincide with Dr. M. Manges, of New York, that heroin in general and glyco-heroin in particular, should exert a favorable effect on all forms of asthma with the exception of one of dyspeptic nature.

*Conclusions*—(1) Cough, though one of many features in the make up of tuberculous affections of the lungs and larynx should, however, receive special attention at the hands of the physician, because it is the most distressing and exhausting factor. (2) Of all the remedies and drugs in our experience, which would tend to ameliorate and suppress cough, we find in glyco-heroin an agent which to all appearances is a remedy par excellence, and we recommend its further trial.

